# New insights into the relationship between taste perception and oral microbiota composition

**DOI:** 10.1038/s41598-019-40374-3

**Published:** 2019-03-05

**Authors:** Camilla Cattaneo, Giorgio Gargari, Ranjan Koirala, Monica Laureati, Patrizia Riso, Simone Guglielmetti, Ella Pagliarini

**Affiliations:** Department of Food, Environmental and Nutritional Sciences (DeFENS), University of Milan, 20133 Milan, Italy

## Abstract

Fairly poor data are available on the relationship between taste perception, food preferences and oral microbiota. In the present study, we investigated the hypothesis that subjects with higher responsiveness to 6-n-propylthiuracil (PROP) might be characterized by a different taste sensitivity and tongue microbiota composition. Indeed, the bacterial metabolism may modulate/enhance the concentration of tastants near the taste receptors, modifying taste perception through a sensorial adaptation mechanism or by a broad range of microbial metabolic pathways. The detection thresholds of sweet, sour, salty and bitter, the Fungiform Papillae Density (FPD) and the composition of bacteria lining the tongue were determined in Supertasters (high PROP responsiveness, ST) and Non-tasters (low PROP responsiveness, NT). An important inter-individual variability was found for all taste stimuli and FPD between the two groups, with NT subjects showing significant higher threshold values and a lower FPD than with STs. We found five bacterial genera whose relative abundances were significantly higher in STs than NTs. This study opens new avenues of research by highlighting associations between parameters usually studied independently.

## Introduction

The contribution of taste perception in individual capacity to recognize the energy and nutrient content of foods and discriminate between safe and poisonous food-substances is well known^1,2^. From birth, people are hard-wired to crave sweet and salty flavours and reject bitter foods^3^. However, later in life, preferences change as a result of repeated food experiences, which can partially explain the great difference in food preferences among human subjects^4^. There is large inter-individual variation in taste perception^5^ and it has been shown that impairments in taste perception and hedonic experience of taste can even cause unhealthy eating habits, which can lead to poor-nutrition or over-nutrition, both representing major public health issues^5^. The most studied and best-understood genetic source of individual variation in oral sensation is 6-n-propylthiouracil (PROP) responsiveness^6–8^, which is influenced by TAS2R38 haplotypes^9^. The TAS2R38 gene is a member of the TAS2R bitter taste receptor gene family. Three single nucleotide polymorphisms (rs714598, rs1726866, rs10246939) at positions encoding amino acids 49, 262 and 296 represent the most common variant alleles of TAS2R38, and encodes two major forms of the PROP receptor, PAV (Proline, Alanine, Valine) and AVI (Alanine, Valine, Isoleucine) haplotypes. Individuals that carry the AVI haplotype (AVI/AVI alleles) are minimally or non-responsive to PROP, while individuals with the PAV haplotype (PAV/PAV alleles or PAV/AVI alleles) demonstrate stronger or intermediate responsiveness^10^. Bartoshuk^11^ expressed the PROP responsiveness as PROP taster status and identified three groups of subjects: PROP Non-tasters (NTs; AVI/AVI alleles), who perceived this compound as weak or tasteless, PROP medium-tasters (MTs; PAV/AVI alleles), who perceived it as moderately bitter, and PROP Super-tasters (STs; PAV/PAV alleles), who perceived it as extremely bitter. PROP responsiveness has long been used as general marker for sensitivity to a variety of sensory stimuli^7^. It has been reported that STs rate the intensity of other bitter compounds, as caffeine and quinine, and other tastants (e.g. salt, sugar, and acid), as more intense than NTs do e.g.^8,12–14^. Moreover, PROP taster status has been associated with greater perception of a variety of orosensory stimuli, including sensations from bitter/astringent fruits and vegetables, fruit juices, and alcoholic beverages compared to NTs^15–17^. It has been suggested that this increased sensitivity could be associated to a greater density of fungiform papillae (FP) located on the tongue, despite data are controversial. Subjects characterized by a greater density of FP (FPD) seem to perceive greater responsiveness when exposed to PROP, sugar, salt and fat creaminess^18–20^.

Since genetic variation in taste receptors may explain some of the observed variability in taste perception, it has been hypothesized that this variability could affect food choice(s) and dietary habits, influencing nutritional and health status, as well as the risk of chronic diseases for a review^21^. In this context, literature data provided mixed results and two major hypotheses were suggested. On one hand, a greater PROP responsiveness seems to be associated with diets rich in saturated fatty acids and added sugars, in contrast to plant-based diets rich in antioxidant and protective phytochemicals generally affecting bitterness of plant foods i.e.^22^. On the other hand, research has also linked higher PROP responsiveness with decreased preferences for high fat and high energy foods and reduced body weight i.e.^23,24^.

Interestingly, it has been suggested that also microbes in the gastrointestinal tract could have a potential direct role in shaping individuals’ eating behaviour and food preferences^25^. The majority of studies of the human microbiota have been focused on the distal gut composition whereas little attention has been paid to microbial communities at other sites along the digestive tract. Notably, a relationship between taste sensitivity and specific oral bacteria has been proposed^26–28^. Particularly, at oral level, papillary structure of the dorsal tongue constitutes one of the major microbial reservoirs of the mouth^29^. However, fairly poor literature about this topic is available and, to our knowledge, the relationship between taste perception and tongue microbiota has not been systematically investigated so far.

In this context, the general aim of the present study was to investigate the relationship among host related factors that are proposed as potential modulators of eating behaviour. In particular, we hypothesized that ST and NT subjects might be characterized by a different taste sensitivity and tongue microbiota composition. Therefore, the orosensory detection thresholds of sweet, sour, salty and bitter, the FPD and the composition of bacteria lining the tongue were determined in these two groups of subjects.

## Results

One hundred and five subjects (52 F and 53 M; age: 23.4 ± 2.5; BMI: 21.9 ± 2.4) were tested in a screening procedure according to their PROP responsiveness. NTs were 28.6% of total sample (n = 30; 15 F and 15 M; age: 24.2 ± 2.8; BMI: 21.6 ± 2.7), whereas STs were 27.6% (n = 29; 17 F and 12 M; age: 22.5 ± 2.1; BMI: 21.5 ± 2.3). The rest of subjects were MTs and were not included in this investigation so that more extreme tasters (super-tasters and non-tasters) could be compared. Thus, only NTs and STs (n = 59, 32 F and 27 M, age: 23.3 ± 2.6 years) were admitted to the main experiment consisting in the assessment of taste sensitivity and oral microbiota composition.

### Taste sensitivity assessment

**Figure 1:**
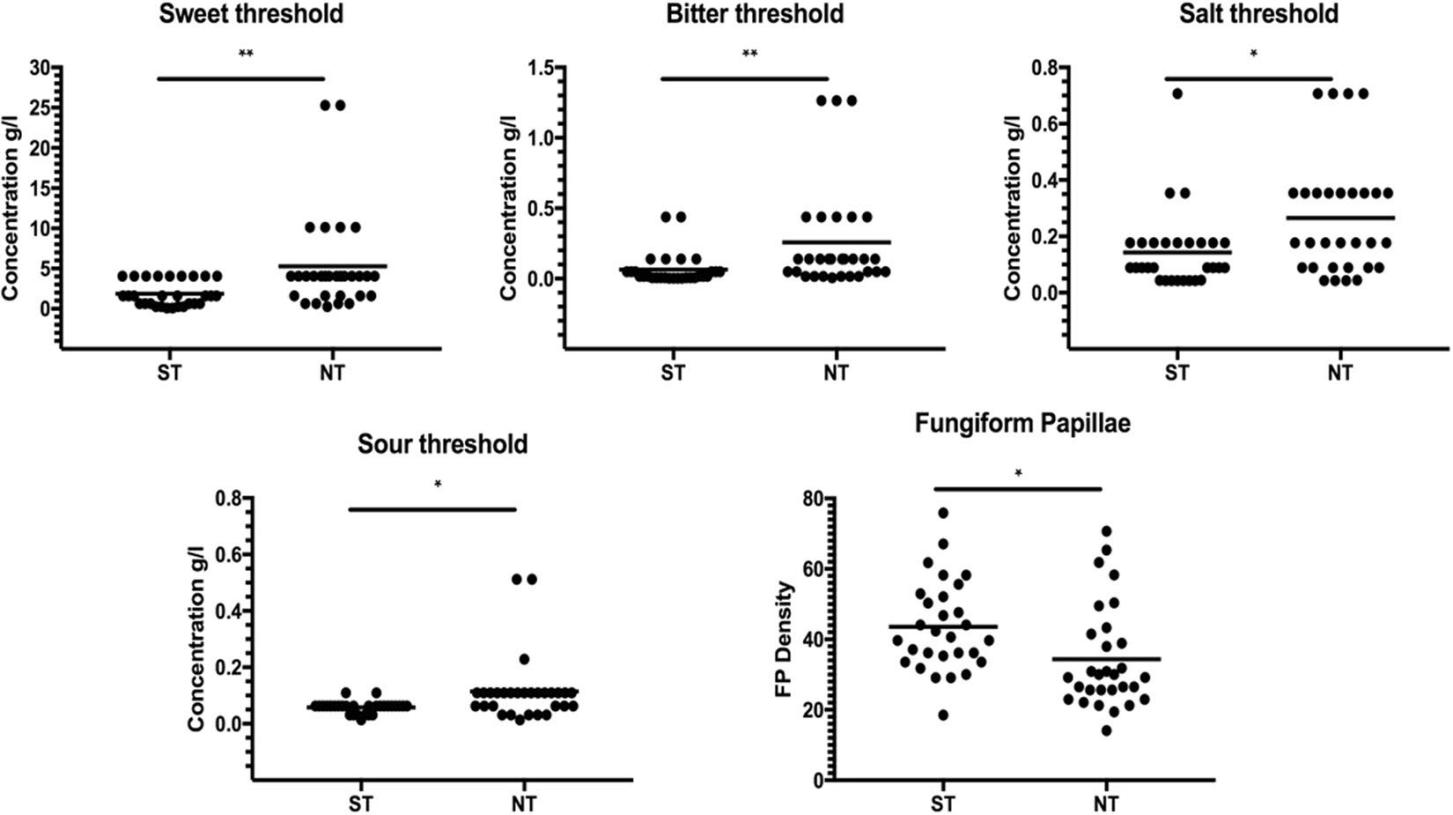
Scatter plots representing the BET of the four-basic taste (sweet, bitter, salt and sour) and the Fungiform Papillae Density (FPD) in super-taster (ST) and non-taster (NT) subjects. Statistics according to unpaired, two tailed Student’s t-test; **p < 0.01; *p < 0.05.

The mean taste threshold values in ST and NT subjects are shown in Fig. 1. An important inter-individual variability was found for all taste stimuli between the two groups, with NTs subjects showing significant higher threshold values (lower sensitivity) compared with STs (sweet taste: t_57_ = 2.90, p = 0.005; salty taste: t_57_ = 2.63, p = 0.011; bitter taste: t_57_ = 2.69, p = 0.009; sour taste: t_57_ = 2.60, p = 0.012). A significant difference was also found in FPD between the two groups of subjects (t_57_ = 2.58, p = 0.013), with NTs subjects showing a significantly reduced FPD compared with STs.

### Characterization of the tongue dorsum microbiota of NT and ST subjects

**Figure 2:**
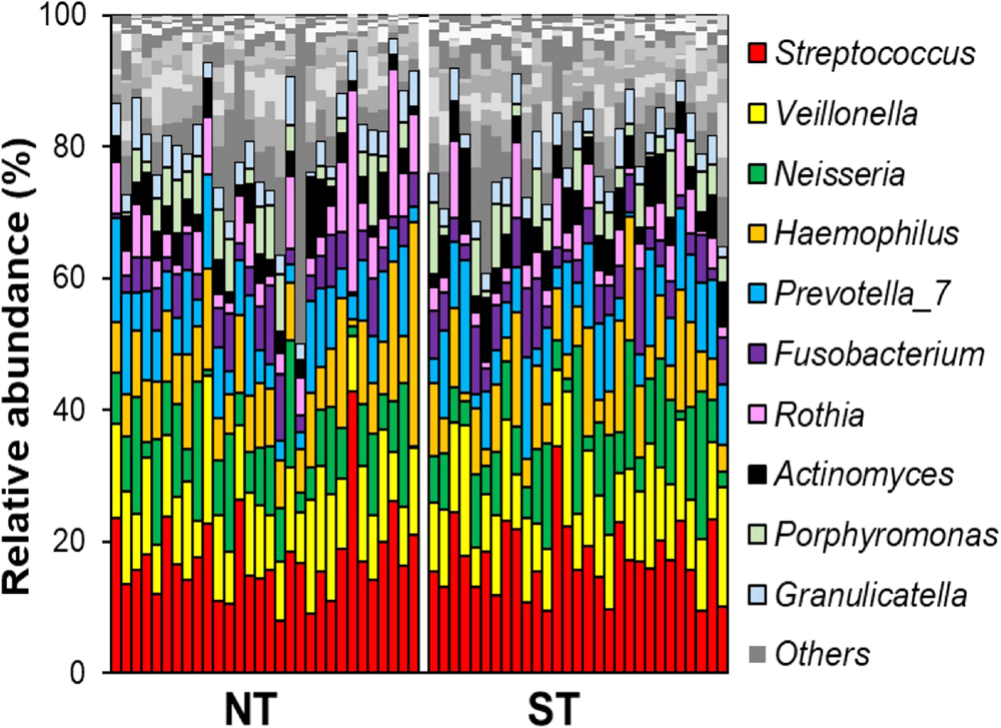
Stacked histograms of bacterial composition in each tongue dorsum sample to the genus level of taxonomic resolution. Each column refers to the bacterial composition in a single sample. NT, non-taster; ST, super-taster. Only the 10 most abundant bacterial genera are shown; other genera are shown in greyscale colour.

To infer possible differences in the tongue microbiota composition between STs and NTs, the DNA extracted from swabs of dorsal tongue surface were analysed by 16S rRNA gene profiling. Intra-sample (α) diversity did not differ significantly between ST and NT samples in term of both taxonomic richness and evenness as calculated through five different indexes (see Supplementary Fig. S1A). In addition, inter-sample (β) diversity measured through UniFrac algorithms did not permit the separation of ST and NT samples (see Supplementary Fig. S1B). At taxonomic level, we identified a total of 141 taxonomic units, with a minimum number of 26 and a maximum of 60 per sample. Overall, 10 taxonomic units (17%; i.e., the genera Streptococcus, Veillonella, Neisseria, Haemophilus, Prevotella, Rothia, Actinomyces, Granulicatella, Alloprevotella, and Gemella) were detected in all 59 samples, and 25 (42%) were found in at least 90% of samples. The bacterial community structure of all analysed samples (determined through DADA2 pipeline, the SILVA ribosomal RNA gene database, and speciateIT taxonomic assignment; “DADA2/SILVA/speciateIT”) was similar and independent from PROP taster status (Fig. 2).

**Figure 3:**
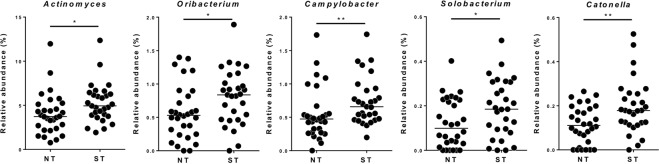
Scatter plots representing the relative abundance of bacterial genera that resulted significantly different between non-taster (NT) super-taster (ST) samples. Statistics according to Mann-Whitney test; **p < 0.01; *p < 0.05.

Nonetheless, at the level of single taxonomic units, we found that five bacterial genera were significantly higher in ST compared to NT samples, namely the Gram-positive *Actinomyces* (belonging to the phylum *Actinobacteria*; P = 0.012 according to Mann-Whitney test), *Oribacterium* (*Firmicutes*; P = 0.034), *Solobacterium* (*Firmicutes*; P = 0.040) and *Catonella* (*Firmicutes*; P = 0.009), and the Gram-negative *Campylobacter* (*Proteobacteria*; P = 0.009) (Fig. 3).

**Figure 4:**
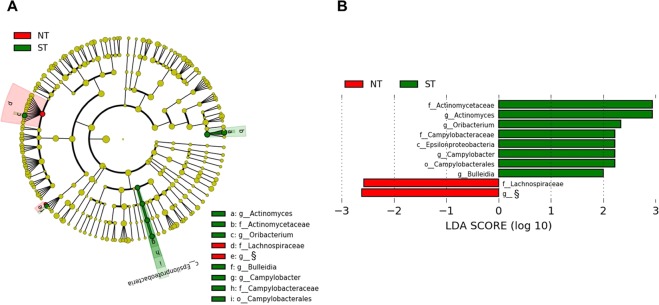
LEfSe analysis of tongue dorsum microbiota in non-taster (NT) and super-taster (ST) subjects. (**A**) Cladogram indicating significantly different taxa (LDA score >2; p < 0.05) at phylum (p_), class (c_), order (o_), family (f_) and genus (g_) levels between NT and ST groups. (**B**) Bar graph displaying LDA scores. Green regions indicate taxa enriched in STs while regions in red indicate taxa enriched in NTs. Differing taxa are listed on the right side of the cladogram. ^§^undefined genus belonging to the family *Erysipelotrichacea*.

Due to the potential impact of the bioinformatic pipeline selected on results in microbiomic analyses, we also compared the microbiota of tongue dorsum of NTs and STs through LEfSe analysis with data generated through QIIME pipeline with Greengenes 16S rRNA gene database; as represented in the resulting cladogram, three genera, i.e. *Actinomyces*, *Oribacterium* and *Campylobacter* were confirmed to be significantly different between the two groups (Fig. 4). In addition, LEfSe analysis found that in ST samples also the *Erysipelotrichaceae* genus *Bulleidia* (*Firmicutes*) was significantly overrepresented, whereas the family *Lachnospiraceae* and an undefined *Erysipelotrichaceae* genus were significantly reduced (Fig. 4). Finally, we also performed Linear Mixed Model analysis to identify potential dependency between the microbiota composition and FPD, also taking into consideration the NT or ST clustering. Using this model, we only observed a trend (p-value 0.059) for the genus *Corynebacterium*.

Taken together, these results indicate that, although the overall community structure of the tongue dorsum microbiota is not dissimilar, specific bacterial taxa with recognized ecological importance at oral level (i.e., the genera *Actinomyces*, *Campylobacter* and *Oribacterium*) are significantly different between NT and ST subjects.

## Discussion

The general aim of the present study was to investigate the relationship among aspects that are proposed as potential modulators of eating behaviour. Over the last decade, the PROP phenotype has received considerable attention for understanding individual differences in taste perception and has also been considered as a marker for food preferences, which could influence dietary behaviour and nutritional status.

Present findings confirmed that STs and NTs differ in their taste ability, with NT subjects showing a significantly lower sensitivity than STs for all tastes. In line with these results, a great number of studies showed that STs rate the bitterness of caffeine as more intense, sucrose as sweeter, sodium chloride as saltier, and citric acid as sourer than NTs do^20,30,31^.

Because the fungiform papillae contain the taste buds of the anterior tongue, it has been suggested that the greater sensitivity of STs subjects could be due to higher FPD^12,23,32–34^. However, this association has not been confirmed in several recent studies^35–40^. The present results support the existence of a relation between PROP sensitivity and FPD and are in line with previous findings which reported that subjects who differ in their response to PROP presented anatomical differences in the tongue.

Considering the above-mentioned literature, in the last decades particular attention has been focused on tongue’s physiology, genetics and related phenotypes in order to provide greater insights into the complexities of human eating behaviour. However, much less attention has been paid to the composition of oral microbiota, which might have an unknown role in taste perception as mouth’s permanent host. Indeed, the papillary structure of the tongue dorsum forms a unique ecological oral site that provides a large surface area for the accumulation of saliva, oral microorganisms and debris^41^. Thus, it appears plausible that oral bacteria lining the tongue may influence and modulate taste perception.

In this context, studies which focused on the identification of specific oral microbial community and its relationship with taste perception are scarce and, generally, did not apply exhaustive methods for the whole taste perception evaluation^27^ or oral microbiota analysis^26^. Solemdal and colleagues^26^ studied variables related to oral health and taste ability in acutely hospitalized elderly. Whole mouth gustatory function was assessed with the “taste strips” method^42^, whereas oral bacteria were assessed with the CRT^®^ Bacteria Kit^43^, which consists cultivation-dependent method for the exclusive determination of *mutans streptococci* and *lactobacilli* in saliva. They found that taste perception (especially for sour) was particularly reduced in acutely hospitalized elderly with high growth of lactobacilli, suggesting that the organic acids produced by bacteria (e.g. lactic, acetic, and propionic acids) may cause adaptation in sour taste perception, and thus increasing the taste threshold for sour. However, this assumption was not supported by microbiomic or predicted metagenome analyses^26^. On the contrary, Besnard and colleagues^27^ applied a microbiomic analysis to study the composition of microbiota and saliva surrounding the circumvallate papillae in combination with the lipid detection threshold in a group of normal weight and obese adults. The multivariate approach highlighted that specific bacteria and salivary signature discriminated between lipid NTs and lipid STs. However, the authors only determined the orosensory detection threshold of linoleic acid (LA) by using the 3-AFC procedure. To our knowledge, our study is the first one that investigated both taste responsiveness and taste detection thresholds for all the basic tastes, applying reliable and sensitive methods, and studied oral microbiota using microbiomic analysis of tongue microbial ecosystem.

In the present study, the analysis of tongue microbiomic profiling data revealed that there was no significant difference in the intra (α)- and inter (β)-subject ecological diversity between ST and NT groups of subjects, confirming previous observations that the tongue dorsum microbiota is characterized by a limited microbial community variation among healthy adults compared to other body sites^44^. The most abundant bacterial groups found in our study are similar to those found in most other studies on healthy subjects. Indeed, 20% of our sequences belonged to the genus *Streptococcus*, confirming the preponderance of this genus within a healthy mouth^45^. Nevertheless, we found that the relative abundance of some taxa was significantly different among STs and NTs. In particular, we identified that major differences exist in five bacterial genera, including the Gram-positive genera *Actinomyces*, *Oribacterium*, *Solobacterium* and *Catonella*, and the Gram-negative *Campylobacter*, which are overrepresented in the STs group.

Moreover, LEfSe analysis confirmed those same taxa to be prevalent as well, but, in addition, showed that the *Erysipelotrichaceae* genus *Bulleidia* was also abundant in STs, whereas the family *Lachnospiraceae* and an undefined *Erysipelotrichaceae* genus were underrepresented.

To infer potential links between bacteria on tongue dorsum and taste responsiveness, we supposed to look into the possibility that bacterial metabolism may modulate/enhance the concentration of tastants near the taste receptors, modifying taste perception through a sensorial adaptation mechanism or by a broad range of microbial metabolic pathways^28,46^. Indeed, it is well known that polysaccharides can be hydrolysed into oligosaccharides, disaccharides, and monosaccharides by host and bacterial glycosidases. In this context, Feng *et al*.^28^ showed a positive correlation between taste sensitivity and some bacterial phyla. In fact, it has been suggested that the presence of *Actinobacteria* and *Bacteroidetes* in the tongue film are linked to an increase sensitivity, especially to bitterness. These bacteria could degrade carbohydrates into disaccharides, monosaccharides and organic acids^47^, which could lead to an enhancement in taste perception near the taste buds. Moreover, many bacteria (e.i. *Actinobacteria*), are known to produce secondary metabolites which are precursors of some bitter acids or bioactive non-nutrient substances, such as phenols, which can enhance the sensation of astringency and the bitter taste in food products^27,48,49^, and cause an adaptation in bitterness and astringency perception. However, the oro-sensory consequences of such changes remain to be determined. Future research is needed using robust analysis on predicted metagenomics data to infer the possibility that some microbial metabolic pathways could discriminate between ST and NT individuals.

In brief, the data reported herein suggest that the microbial composition of the tongue microbiota of people with higher taste responsiveness are different from those of people of a reduced taste responsiveness, and provide new references that these differences of oral bacteria lining the tongue may influence and modulate taste perception. Corroboration of these results using a larger sample size of ST and NT subjects and a deepen study of subjects’ eating habits might achieve a better understanding into several aspects, considered as potential modulators of eating behaviour. Nevertheless, our results offer new insights into the reciprocal impact between the oral microbiota and taste perception. Based on an analysis of tongue microbiota, taste responsiveness and detection threshold of the four-basic taste, this study opens new avenues of research by highlighting associations between parameters usually studied independently.

## Material and Methods

### Participants

One hundred and five normal-weight young adults were recruited from the University community (i.e. through public advertisement). Individuals were excluded if they were pregnant or lactating women, had medical conditions, treatments that could modify taste perception or were habitual smokers. Moreover, subjects who consumed any medication, probiotics or antibiotics two months before the study were also excluded. Habitual use of mouthwash was also considered a criterion for volunteer exclusion.

Informed, written consent was obtained from all subjects on the first test day. The present study was performed according to the principles established by the Declaration of Helsinki and the protocol was approved by the Institutional Ethics Committee of the University of Milan.

### Procedure

Subjects were instructed to refrain from ingestion of all foods, beverages, and oral care products for a minimum of 3 h before arrival to the laboratory. All testing was completed in 2 test sessions. Subjects were familiarized with all procedures and rating scales at the start of the first session, when a screening procedure with PROP solution was performed. Participants (n = 105) were selected according to their thiourea taste sensitivity (PROP status) and they required to be NTs or STs. Then, if a subject was identified as NT or ST was admitted to the second session, in which microbiota sampling and taste threshold were evaluated. FP were also counted at this time.

### Screening procedure

A method proposed by Prescott and colleagues^31^ was used as an initial screen for PROP status. The intensity of bitterness of a supra-threshold 3.2 mmol/L solution of PROP (European Pharmacopoeia Reference Standard, Sigma-Aldrich, Milano, Italy) was rated using the Generalized Labeled Magnitude Scale (0–100), gLMS^50^. Subjects were presented with 2 identical samples (10 ml) coded with a three-digit number and were instructed to hold each sample (10 ml) in their mouth for 10 s, then to expectorate the solution and wait 20 s before evaluating the intensity of bitterness. In order to control for carry-over effect after the first sample evaluation, subjects had a 90 s break to rinse their mouths with water. The average bitterness score was used for each subject.

Respondents were grouped according to their PROP status based on arbitrary cut-offs as proposed by Laureati and colleagues^51^. Participants were categorized as NTs if they rated the PROP solution lower than 17 mm on the gLMS, whereas they were categorized as STs if they rated the PROP solution higher than 53 mm on the gLMS. According to previous studies the Medium-tasters (MTs) were not included in this investigation so that more extreme tasters (super-tasters and non-tasters) could be compared^52–54^.

### Taste sensitivity evaluation

#### Stimuli

Tastants were sucrose, sodium chloride, citric acid, and caffeine (Sigma-Aldrich) dissolved in mineral water (Levissima, San Pellegrino spa), representing the four basic tastes - sweetness saltiness, sourness, and bitterness, respectively. For each taste 7 concentrations were prepared in successive dilutions. The total range of concentrations was chosen on the basis of threshold values reported in the literature^55–57^ and were adjusted according to preliminary tests. Concentration ranges were established such that the lowest concentration was clearly below and the highest concentration was clearly above the level at which subjects could detect or recognize the stimulus. This resulted in the following ranges of tastants in water in g/l: sodium chloride 6.25 × 10^−2^–4 (0.4 log steps); sucrose 1.6 × 10^−1^–40 (0.4 log steps); citric acid 2 × 10^−2^–1.5 (0.3 log steps); caffeine 3 × 10^−3^–2 (0.4 log steps). The solutions were tested at room temperature and kept at 5 °C in the dark for no longer than 2 days.

#### Taste threshold assessment

Taste thresholds were evaluated using the 3-AFC (Alternative Forced Choice) method reported in ISO/DIS 13301:2018^58^. This international standard describes a reliable procedure to estimate the value of a threshold for any stimulus presented in an aqueous medium. For each stimulus, subjects were presented with 7 triads of samples coded with a three-digit number. Each triad consisted of 1 sample containing the stimulus and 2 identical samples (10 ml) of a blank solution (mineral water). The 7 triads proceeded from a weaker to an increasingly stronger concentration, and the position of the sample containing the stimulus was randomized over trials and assessors. For each triad, participants were instructed to select the sample which was different from the other 2^59^. If the assessors were uncertain, they were instructed to guess (forced choice procedure). At the beginning of each session, and before each triad, the assessors were instructed to rinse their mouth with mineral water.

#### Fungiform papillae density (FPD) assessment

The individual FPD was calculated following the procedure previously described by Monteleone and colleagues^60^. In brief, the tongue was swabbed with household blue food colouring, using a cotton-tipped applicator, to make fungiform papillae (FP) easily visible on the anterior portion of the dorsal surface of the tongue. Digital pictures were recorded using a digital microscope (MicroCapture, version 2.0 for 20x–400x) and the clearest image was selected. Then, the number of FP was counted in two 0.6 cm diameter circles, one on right side and one on left side of tongue, 0.5 cm from the tip and 0.5 cm from the tongue midline, following the Denver Papillae Protocol^58^. The average of these values was used for each subject. The individual FPD was then calculated by reporting the number of FP to a common unit area of 1 cm^2^.

### Microbiomic evaluation

#### Oral sample collection and DNA Extraction

Volunteers were restricted for at least 3 h of food intake prior to sample collection as mentioned previously. The following instructions for self-tongue swab collection were given: volunteers were asked to sit in front of a mirror, bulge out the tongue and gently press the swab on the surface rolling and touching edges, tip and all defined area of the tongue (about 2/3 of the length) for two minutes using a sterile flocked swab (FLOQSwabs^TM^, COPAN S.p.A., Brescia, Italy). The swab samples were immediately placed in 750 µl of Power Bead solution provided in the DNeasy PowerLyzer PowerSoil DNA extraction kit (Qiagen, Hilden, Germany) and stored at −80 °C. For DNA extraction, samples were thawed on ice and homogenized for five minutes to release all the bacterial cells in the solution. Then, swabs were dried pressing several times on the interior wall of the tube. Finally, samples were processed by means of the DNA extraction kit mentioned above following manufacturer’s instructions with a minor modification consisting of incubating samples at 65 °C for 10 min after addition of C1 solution. Mechanical bacterial cell disruption has been performed using a Precellys bead beater kept in a cold room (3 cycles of 6800 rpm × 30 s; Advanced Biotech Italia s.r.l., Seveso, Italy). Quantification and verification of the 260/280 ratio of the extracted DNA was carried out with a Take3 Micro-Volume plate in a Gen5 microplate reader (BioTek Instrument Inc., Winooski, VT, USA). Finally, DNA samples were stored at −80 °C.

#### Tongue microbiota analysis

The DNA extracted from tongue swabs was analysed at the Institute for Genome Sciences (University of Maryland, School of Medicine, Baltimore, MD, USA) through 16 S rRNA gene profiling with Illumina HiSeq 2500 rapid run sequencing of the V3–V4 variable region. Sequencing reads were analysed following a pipeline comprehensive of two main steps: (i) pairing and filtering of raw amplicon sequencing data by DADA2 (R package); (ii) taxonomic assignment of each amplicon sequence with speciateIT on SILVA database according to the custom pipeline freely available on GitHub (https://github.com/Ravel-Laboratory/speciateIT). A total of 1,938,469 filtered high-quality sequence reads were generated with a mean ± standard deviation (SD) of 32,855 ± 21494 reads per sample; maximum of 69359 for sample C55 and minimum of 808 for sample C26. The negative control introduced internally to the entire process gave 187 reads (183 ascribed to *Lactobacillus gasseri* and 4 to *Lactobacillus reuteri*). Analysis and taxonomic assignment of sequencing reads were also performed by means of the bioinformatic pipeline Quantitative Insights Into Microbial Ecology (QIIME) version 1.9.0^61^ with the GreenGenes database (version 13_5). Metadata have been deposited in the European Nucleotide Archive (ENA) of the European Bioinformatics Institute under accession code PRJEB28769.

### Statistical analysis

The matrix of the correct and incorrect answers produced separately by each judge was used to calculate the individual taste threshold. The individual’s Best Estimate Threshold (BET) for each sensory stimulus was calculated as the geometric mean of the highest concentration missed and the next higher concentration that was correctly recognized (ISO/DIS 13301:2018). After verifying that taste sensitivity data (taste thresholds and FPD) were normally distributed, the differences between two groups (STs vs NTs) were assessed using an unpaired, two tailed Student’s t-test using IBM SPSS statistical software version 25 (SPSS Inc, Chicago, IL, USA). For microbiomic data, significant differences between NT and ST were determined according to Mann-Whitney test with Benjamini-Hochberg correction. In addition, microbial composition differences between groups have been defined with QIIME/Greengenes-generated data through LDA Effect Size (LEfSe)^62^. Linear mixed model was carried out as regression analysis between the microbiota and FPD factors taking into account the binary clustering NT/ST. p < 0.05 was considered to be significant and the range 0.05 ≤ p < 0.10 was accepted as a trend. For microbiomic data, statistical calculations were performed using the software program GraphPad Prism 5.

## Supplementary information

Supplementary material

## Data Availability

The datasets generated during and/or analysed during the current study are available from the corresponding author on reasonable request.
