# Supplementary material for: New insights into the relationship between taste perception and oral microbiota composition
Source: Sci Rep. 2019 Mar 5;9:3549. doi: 10.1038/s41598-019-40374-3 (PMC6401163; doi:10.1038/s41598-019-40374-3)
Supplement: Supplementary file 1 — Supplementary material [file 41598_2019_40374_MOESM1_ESM.pdf]

New insights into the relationship between taste perception and oral microbiota composition

Camilla Cattaneo*, Giorgio Gargari, Ranjan Koirala, Monica Laureati, Patrizia Riso, Simone Guglielmetti°, Ella Pagliarini°

Department of Food, Environmental and Nutritional Sciences (DeFENS), University of Milan, 20133 Milan, Italy

**Correspondence to*: Camilla Cattaneo, Department of Food, Environmental and Nutritional Sciences (DeFENS), University of Milan, 20133 Milan, Italy
e-mail: camilla.cattaneo@unimi.it
Telephone: +39 0250319175

° contributed equally to the work and must be considered as co-last authors

Supplementary material

Fig. S1. Diversity analyses of the microbiota composition of tongue dorsum in PROP super-taster (ST) and non-taster (NT) subjects. **A**, intra-sample diversity as determined through five different α-diversity; **B**, β-diversity analyzed through weighted and unweighted UniFrac.

A

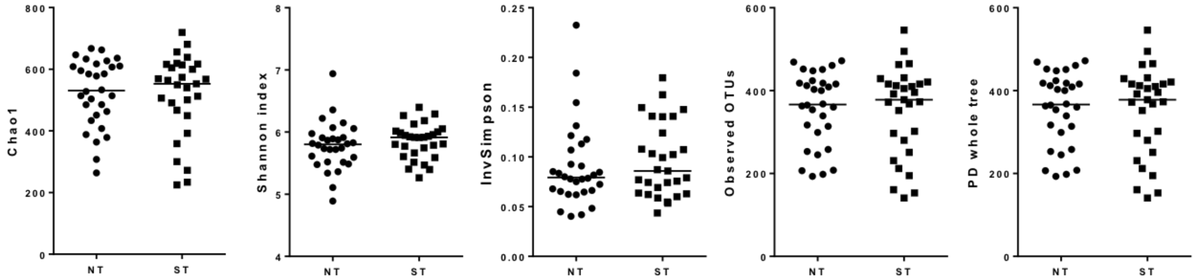

B

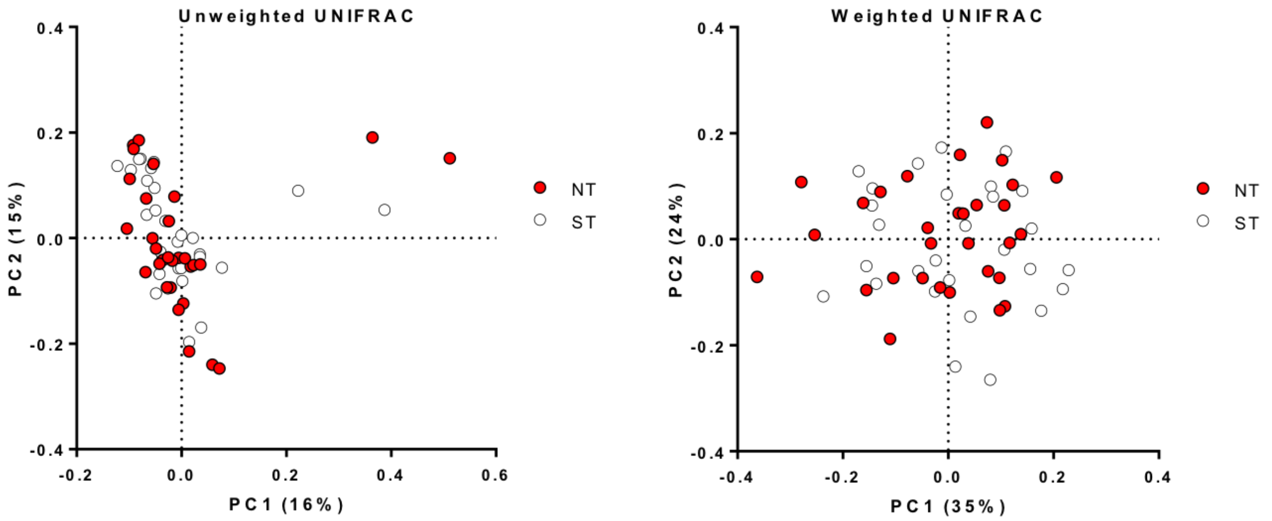

Tab. S1. Number of reads per sample after filtering

Sample	nr. of reads
C01	30293
C02	29367
C03	69359
C04	46565
C05	54926
C06	4897
C07	56247
C08	35973
C09	2394
C10	11381
C11	59968
C12	4054
C13	50651
C15	24255
C16	53572
C17	3195
C18	50395
C19	39925
C20	34501
C21	46723
C22	10267
C23	34424
C24	52696
C25	3032
C26	808
C27	814
C28	68470
C29	43714
C30	45225
C31	47584
C32	12408
C33	66066
C34	27485
C35	36198
C36	987
C37	5665
C38	8094
C39	51913
C40	53132
C41	54532
C42	51846
C43	54572

C44	67383
C45	22440
C46	41566
C47	39163
C48	27742
C49	35895
C50	51456
C51	34603
C52	23132
C53	9041
C54	8662
C55	64944
C56	27864
C57	33872
C58	2253
C59	2117
C60	7763
